# Correlation of TRPA1 RNAscope and Agonist Responses

**DOI:** 10.1369/00221554241251904

**Published:** 2024-05-10

**Authors:** Natalia S. Rojas-Galvan, Cosmin I. Ciotu, Stefan Heber, Michael J.M. Fischer

**Affiliations:** Centre for Physiology and Pharmacology, Medical University of Vienna, Vienna, Austria and Randall Centre for Cell & Molecular Biophysics, King’s College London, London, UK; Centre for Physiology and Pharmacology, Medical University of Vienna, Vienna, Austria; Centre for Physiology and Pharmacology, Medical University of Vienna, Vienna, Austria; Centre for Physiology and Pharmacology, Medical University of Vienna, Vienna, Austria

**Keywords:** dorsal root ganglion, in situ hybridization, RNAscope, TRPA1, TRPV1

## Abstract

The TRPA1 ion channel is a sensitive detector of reactive chemicals, found primarily on sensory neurons. The phenotype exhibited by mice lacking TRPA1 suggests its potential as a target for pharmacological intervention. Antibody-based detection for distribution analysis is a standard technique. In the case of TRPA1, however, there is no antibody with a plausible validation in knockout animals or functional studies, but many that have failed in this regard. To this end we employed the single molecule in situ hybridization technique RNAscope on sensory neurons immediately after detection of calcium responses to the TRPA1 agonist allyl isothiocyanate. There is a clearly positive correlation between TRPA1 calcium imaging and RNAscope detection (*R* = 0.43), although less than what might have been expected. Thus, the technique of choice should be carefully considered to suit the research question. The marginal correlation between TRPV1 RNAscope and the specific agonist capsaicin indicates that such validation is advisable for every RNAscope target. Given the recent description of a long-awaited TRPA1 reporter mouse, TRPA1 RNAscope detection might still have its use cases, for detection of RNA at particular sites, for example, defined structurally or by other molecular markers.

## Introduction

The Transient Receptor Potential channel subtype ankyrin 1 (TRPA1) is activated by several chemical compounds, electrophilic and non-electrophilic, thus serving the role of sensing potentially noxious molecules.^1,2^ TRPA1 functions as a nonselective cation channel with a particularly high permeability for calcium. It is mainly expressed by nociceptive primary sensory neurons, the somata of which are located in the dorsal root ganglia (DRG), trigeminal ganglia and nodose ganglia.^3,4^ Although patterns might have somewhat changed with the advent of single-cell RNAseq, TRPA1 expression in about half of the neurons expressing transient receptor potential vanilloid 1 (TRPV1) is a reasonable estimate.^5^ Neurons expressing TRPV1 are commonly considered nociceptive, but within these, no difference in sensation was observed when evaluating the TRPA1-expressing half compared with activation of all TRPV1-expressing neurons.^6^ After detection in cultured fibroblasts,^7^ TRPA1 was reported on other neuronal and non-neuronal cell types.^8–11^ Recently, a TRPA1 reporter mouse strain indicates a more limited expression than antibody-based studies have suggested.^9^ We have challenged that TRPA1 and TRPV1 are functional in cardiomyocytes.^12,13^ This implies, beyond the heart, that the functional expression of these two channels should be reconsidered, particularly when reports on different cell types were based on pharmacological tools and antibodies with limited specificity. The detection of TRPA1 based on antibodies is especially problematic, in both blots and immunohistochemistry, mainly due to the poor specificity of most of these antibodies. Antibody-based detection is one of the standard techniques used to investigate protein expression. However, the majority of the most commonly used antibodies to detect human TRPA1 were invalidated.^14^ This has led to a situation where it requires a tedious check whether there is a functional expression in a particular tissue or cell type. On RNA level, the in situ hybridization based technique known as RNAscope ^15^ has improved the sensitivity of RNA-based fluorescence in situ hybridization (FISH) to single RNA molecule visualization with high specificity. Based on sensitivity and specificity, RNAscope has been judged a reliable and robust method that could complement established clinical diagnostics.^16^ A major caveat of RNAscope-based gene expression studies is the potential discordance between mRNA and functional protein presence. In this respect, we favor functional validation over immunohistochemistry in case reliable antibodies are missing, as immunohistochemistry is usually a surrogate marker for a functional channel, just as the RNA is a surrogate for the presence of protein. The aim of this study is to evaluate the correlation between RNAscope and functional responses of TRPA1.

## Materials and Methods

### Chemicals and Solutions

The extracellular solution used for cellular experiments contains (in mM): 145 NaCl, 5 KCl, 10 glucose, 10 4-(2-Hydroxyethyl)piperazine-1-ethanesulfonic acid (HEPES), 1.25 CaCl_2_, and 1 MgCl_2_ (Merck), buffered to pH 7.4 with NaOH and has an osmolarity of 300 mosmol. Salts and HEPES were obtained from Carl Roth (Karlsruhe, Germany), Sigma-Aldrich (St. Louis, MO) or Merck (Darmstadt, Germany), NaOH from Thermo Fisher Scientific (Waltham, MA). Allyl isothiocyanate (AITC) and capsaicin were obtained from Sigma-Aldrich.

### Isolation of Primary Dorsal Root Ganglion Neurons

Breeding, euthanasia and all procedures of animal handling were performed according to regulations of animal care and welfare. Experiments were carried out in accordance with the European Communities Council Directive of November 24, 1986 (86/609/EEC). Wild type C57BL/6 mice were anesthetized by exposure to isoflurane or rising CO_2_ levels and euthanized by cervical dislocation. Dorsal root ganglias from all spinal levels were excised and transferred to DMEM (D5648, Sigma-Aldrich) containing streptomycin/penicillin 1% and L-glutamine 1% (Lonza, Basel, Switzerland), treated with 1 mg ml^-1^ collagenase (Sigma-Aldrich) and 3 mg ml^-1^ Dispase II (Roche Applied Science, Penzberg, Germany) for 55 min at 37°C. Digested DRGs were then mechanically dissociated with a Pasteur pipette, centrifuged at 1200 rpm for 5 min and plated onto 35 mm glass-bottomed dishes or 12 mm glass coverslips previously coated with poly-D-lysine (100 mg ml^-1^, Sigma-Aldrich). Dorsal root ganglia neurons were cultured in DMEM with streptomycin/penicillin and l-glutamine and in addition supplemented mouse nerve growth factor 100 ng ml^-1^ (Alomone Labs, Tel Aviv, Israel). Neurons were cultured at 37°C and 5% CO_2_ for 15 to 30 hr.

### Single-Cell Fluorescent Imaging

Due to the high calcium permeability of TRPA1,^17^ observation of cytosolic calcium by microfluorimetry is a method of choice. The coverslips were incubated with Fura-2 AM ester (Biotium, Fremont, CA) for 30 min at 37°C and 5% CO_2_, before placement in glass-bottom 35 mm dishes in extracellular solution and a recovery period of 10 min. Dishes were placed in an Olympus IX73-inverted microscope (Olympus, Tokyo, Japan) and imaged using a 10× objective. Cells were permanently superfused with extracellular solution using a software-controlled 8-channel, gravity-driven, common-outlet system (ALA Scientific Instruments Inc, Farmingdale, NY). A positive control to detect viable cells was added at the end of each recording by depolarization through an extracellular solution containing 60 mM KCl, isotonically replacing NaCl. Fura-2 was alternatingly excited for 30 ms by a 340 nm LED (50 mW, used at 100%) and by a 385 nm LED (1435 mW, used at 5%) using an Omicron LEDHub (Laserage-Laserprodukte GmbH, Rodgau-Dudenhofen, Germany). Fluorescence emission was long-pass filtered at 495 nm, and pairs of images were acquired at a rate of 1 Hz with a 4.2 megapixel 16 bit CCD camera (6.5-μm pixel edge length, 18.8 mm sensor diameter, Prime BSI; Teledyne Photometrics, CITY, AZ). The hardware was controlled by the μManager 1.4 plugin in ImageJ.^18^ The background intensity was subtracted before calculating the ratio between the fluorescence emitted when the dye was excited at 340 nm and at 385 nm (F340/F385). The time course of this ratio was analyzed for regions of interest adapted to individual cells. The areas under the curve were calculated for a period of 60 sec after the start of each agonist application and baseline corrected using the average of the respective region of interest over a period of 5 sec, ending 3 sec before the beginning of the application.

### HEK293t Cells and Transient Transfection

HEK293t cells were cultured in DMEM supplemented with fetal bovine serum 10% (Sigma-Aldrich), streptomycin/penicillin 1% and L-glutamine 1% (Lonza), at 37C and 5% CO_2_. HEK293t cells were transiently co-transfected with JetPEI reagent (PolyPlus, Illkirch, France) using pcDNA3.1 encoding for mouse TRPA1 and a CFP-YFP fusion (CFP::YFP, non-flexible 16 amino acid linker; the construct can also be used as positive control for FRET assays, pEGFP-c1 backbone) in a 1:4 to 1:2 ratio. For mock transfections, pcDNA3.1 encoding for the prostaglandin EP1 receptor was used.

### RNAscope in situ hybridization

Dorsal root ganglia neurons were fixed immediately after single-cell fluorescent imaging with paraformaldehyde 4% for 30 min at room temperature. HEK293t were fixed 24 hr after seeding. Paraformaldehyde 4% was removed and following three washes with PBS 1x, cells were dehydrated in 50% ethanol (1 min), 70% ethanol (1 min), 100% ethanol (1 min) at room temperature. The Petri dishes were air-dried and circular hydrophobic barriers were drawn surrounding the cells with a hydrophobic PAP barrier pen (ImmEdge, Vector Laboratories, England, UK). Cells were rehydrated in 100% ethanol (1 min), 70% ethanol (1 min), 50% ethanol (1 min) and permeabilized with PBS 1x + 0.1% Tween 20 (10 min). RNAscope was performed with probes and a kit following manufacturer’s instructions (Advanced Cell Diagnostics, Newark, CA). This includes treatment with hydrogen peroxide (10 min), and then with Protease III diluted at 1:15 (10 min), as provided with the kit. RNAscope Multiplex Fluorescent V2 assay was performed with probes against mouse TRPV1 (Mm-Trpv1, 313331) and mouse TRPA1 (Mm-Trpa1-C2, 400211-C2). Opal dyes 570 and 690 (Akoya Biosciences, Marlborough, MA) were used at 1:1500 dilution to detect the corresponding probes. RNA quality was checked with a positive probe mix (320881) against RNA polymerase II subunit A (PolR2A-C1) and Peptidylprolyl Isomerase B (PPIB-C2). Non-specific signal and background were detected with a negative control probe (320871) that targets the bacterial gene dihydrodipicolinate B. subtilis reductase (DapB).

### Confocal Imaging and Image Alignment

Fluorescent images were acquired with a confocal microscope (Nikon Eclipse Ti A1, Tokyo, Japan) with laser excitation wavelengths of 405 nm, 487 nm and 640 nm, with the 10× objective with 0.45 NA for correlation to functional data, and the 60× objective with 1.4 NA for the individual cell including neurites, at a pinhole of 25.5 µm. Z-series with 21-25 optical planes and Z-steps of 0.5 μm were used for cultured DRGs; 1 μm steps for HEK293t cells. The transmitted light images were acquired simultaneously with the fluorescent imaging, with bottom illumination from the coherent laser light source; transmitted light was focused by a collecting lens onto an additional PMT located above the sample. Image analysis was performed with ImageJ. For correlation to functional measurements, Z-stacks were combined by average intensity projection, as the latter reflects multiple signals in the path of light. In contrast, for detection of RNAscope in axons, a maximum intensity projection was used. Alignment was performed manually within the TrakEM 2 plugin for ImageJ^19^ by applying rigid transformations on the transmission image acquired with the confocal microscope until it matched the transmission image acquired with the wide field microscope. Rotation, translation, and scaling parameters were recorded and propagated to the other channels (immunofluorescence for TRPA1 and TRPV1 RNAscope as well as DAPI staining).

### Data analysis

It was tested whether the RNAscope signal intensity as an index for the amount of TRPA1 mRNA and the functional response to AITC 100 µM of the same neuron correlate. All cells with an above-threshold signal on either RNAscope or functional response were considered. For the area under the curve, a Gaussian distribution was fitted to the values of cells without response, mean + 2*SD was defined as the threshold for a response. Similar, for RNAscope, mean + 2*SD of the intra-day negative control was defined as threshold. A Pearson product-momentum correlation was calculated. A similar analysis was performed for TRPV1.

## Results

### ISH Detects Murine TRPA1 and TRPV1 in Cultured Cells

**Figure 1.**
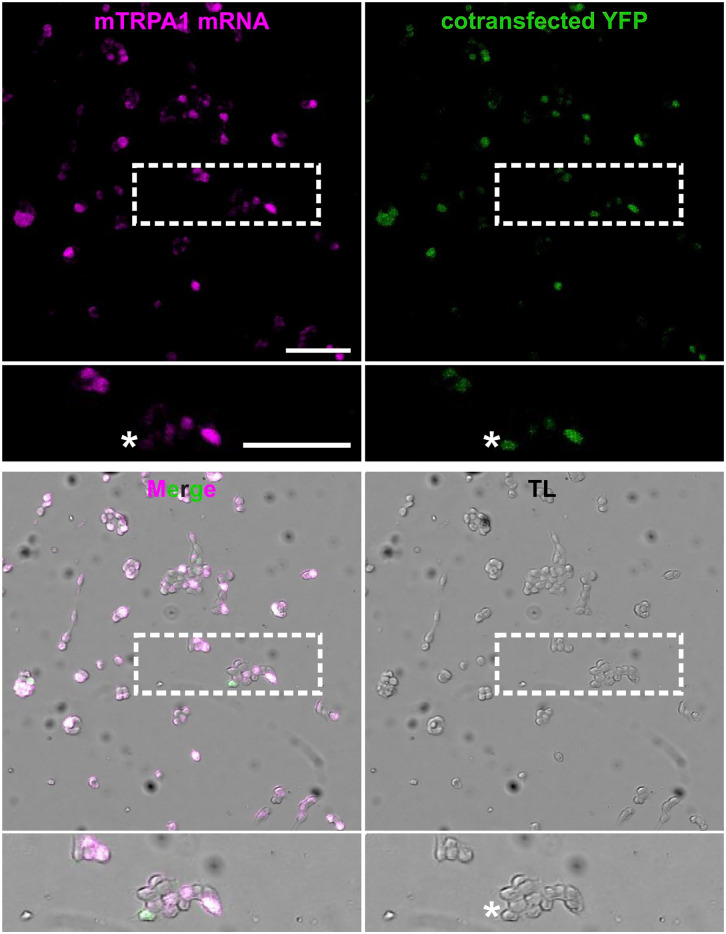
RNAscope detects TRPA1 mRNA in transfected HEK293t cells. Representative images of mTRPA1 mRNA (magenta), YFP (green), transmitted light (TL) or the overlay of these images in HEK293t cells co-transfected with mouse TRPA1 and CFP::YFP (ratio 1:2). Enlarged image of cells (dashed white box) allows a detailed observation of colocalization of magenta and green signals in co-transfected HEK293t cells. Size and transfection efficacy of the cDNAs might explain cells with a YFP but without RNAscope signal (white asterisk). Scale bars = 100 μm.

**Figure 2.**
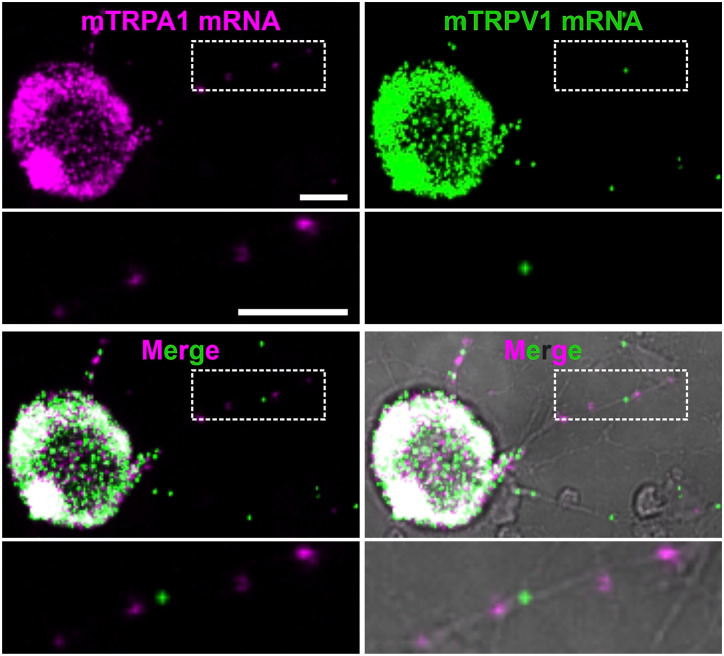
TRPA1 and TRPV1 mRNA are detected in cell bodies and axons of cultured sensory neurons. Representative images of TRPA1 mRNA (magenta), TRPV1 mRNA (green), the overlay of TRPA1 and TRPV1 mRNA and the overlay with transmitted light (gray) in 24 hr cultured mouse DRGs. Enlarged image (dashed white box) shows the presence of TRPA1 and TRPV1 mRNA punctae in neurites. Scale bar = 10 μm.

**Figure A1.**
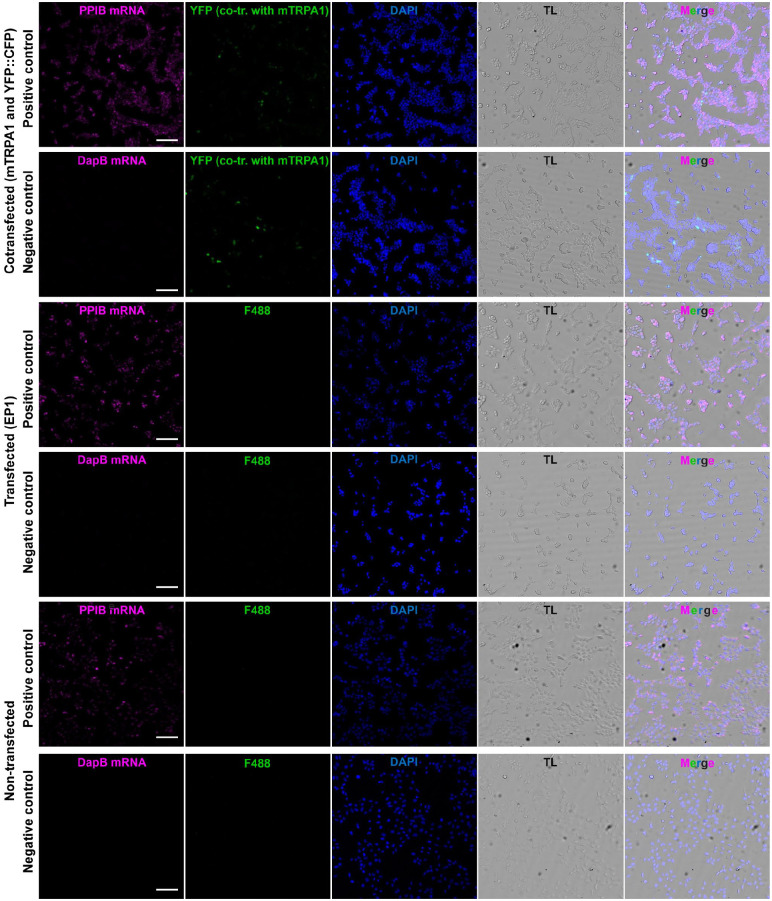
RNA quality check in HEK293t cells. Representative images of positive and negative controls in (from top to bottom) HEK293t cells co-transfected with mTRPA1 and CFP::YFP, HEK293t cells transfected with prostaglandin EP1 receptor and non-transfected HEK293t cells. Positive control for RNA integrity and fluorescent signal detection is Peptidylprolyl Isomerase B mRNA (PPIB) (magenta), the bacterial gene DapB mRNA (magenta) serves as negative control. YFP (green), DAPI (blue), transmitted light (TL, gray) and the overlay of these images with positive and negative controls are also depicted. For HEK293t cells that were not transfected with CFP::YFP, the fluorescence emitted upon excitation wavelength of 488 nm (F488) (green) was included. All scale bars = 100 μm.

**Figure A2.**
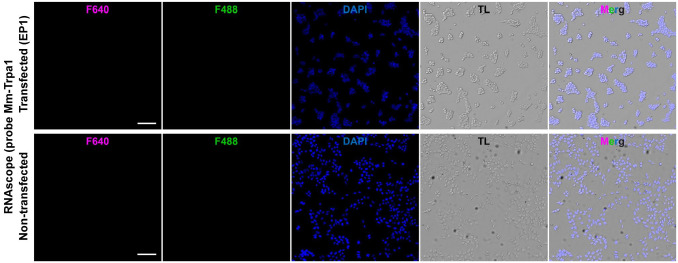
TRPA1 mRNA is not detected in mock- or non-transfected HEK293t. Representative image of fluorescence emitted with excitation wavelength of 640 nm (F640) (magenta), fluorescence emitted with excitation wavelength of 488 nm (F488, green), DAPI (blue), transmitted light (TL, gray) and the overlay of these images in HEK293t cells transfected with prostaglandin EP1 receptor (top) and non-transfected HEK293t cells (bottom). Scale bars = 100 μm.

**Figure A3.**
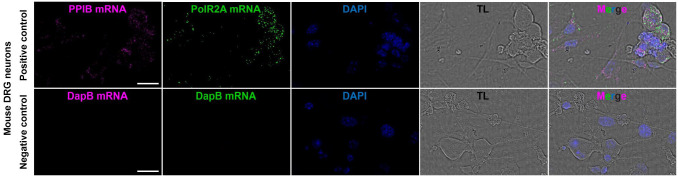
RNA quality check in mouse DRGs. Representative image of positive (top) and negative (bottom) controls in mouse DRG neurons. Positive controls for RNA integrity are Peptidylprolyl Isomerase B mRNA (PPIB) (top magenta) and RNA polymerase II subunit A (PolR2A) (top green). Negative control for fluorescent signal detection is the bacterial gene DapB mRNA in two channels (bottom magenta and bottom green). DAPI (blue), transmitted light (TL) and the overlay of these images are also depicted. Scale bars = 20 μm.

RNAscope performance to detect mouse TRPA1 (mTRPA1) mRNA was tested in HEK293t cells co-transfected with mTRPA1 and CFP::YFP (Fig. 1A). In total, from 724 cells, 527 (73%) were TRPA1 and YFP positive, while 197 (27%) were YFP positive and only 2 were TRPA1 positive. This suggests a specific detection of TRPA1 mRNA. The smaller plasmid size and the larger amount of CFP::YFP cDNA compared with mTRPA1 might explain the observed co-transfected HEK293t cells that had only YFP fluorescence. To support the detection of mTRPA1 mRNA in sensory neurons, cultured DRG neurons were co-stained by ISH with probes for mTRPV1 and mTRPA1 ion channels (Fig. 2). Colocalization of TRPA1 and TRPV1 mRNA molecules was observed. In neuronal cell bodies mRNA signal was so high that individual molecules were not fully discernible. Single mRNA molecules could be detected in neurites of cultured DRG neurons, and TRPA1 and TRPV1 single mRNA molecules were localized in the same neurites. Positive and negative controls for ISH were performed to ensure a high level of RNA integrity and the absence of false-positive fluorescent signals in HEK293t cells (Fig. A1). Mock transfections were used as additional negative controls (Fig. A2). Positive and negative controls were also performed in sensory neurons (Fig. A3).

### RNAscope Detects Functional TRPA1 and TRPV1 in Cultured DRG Neurons

**Figure 3.**
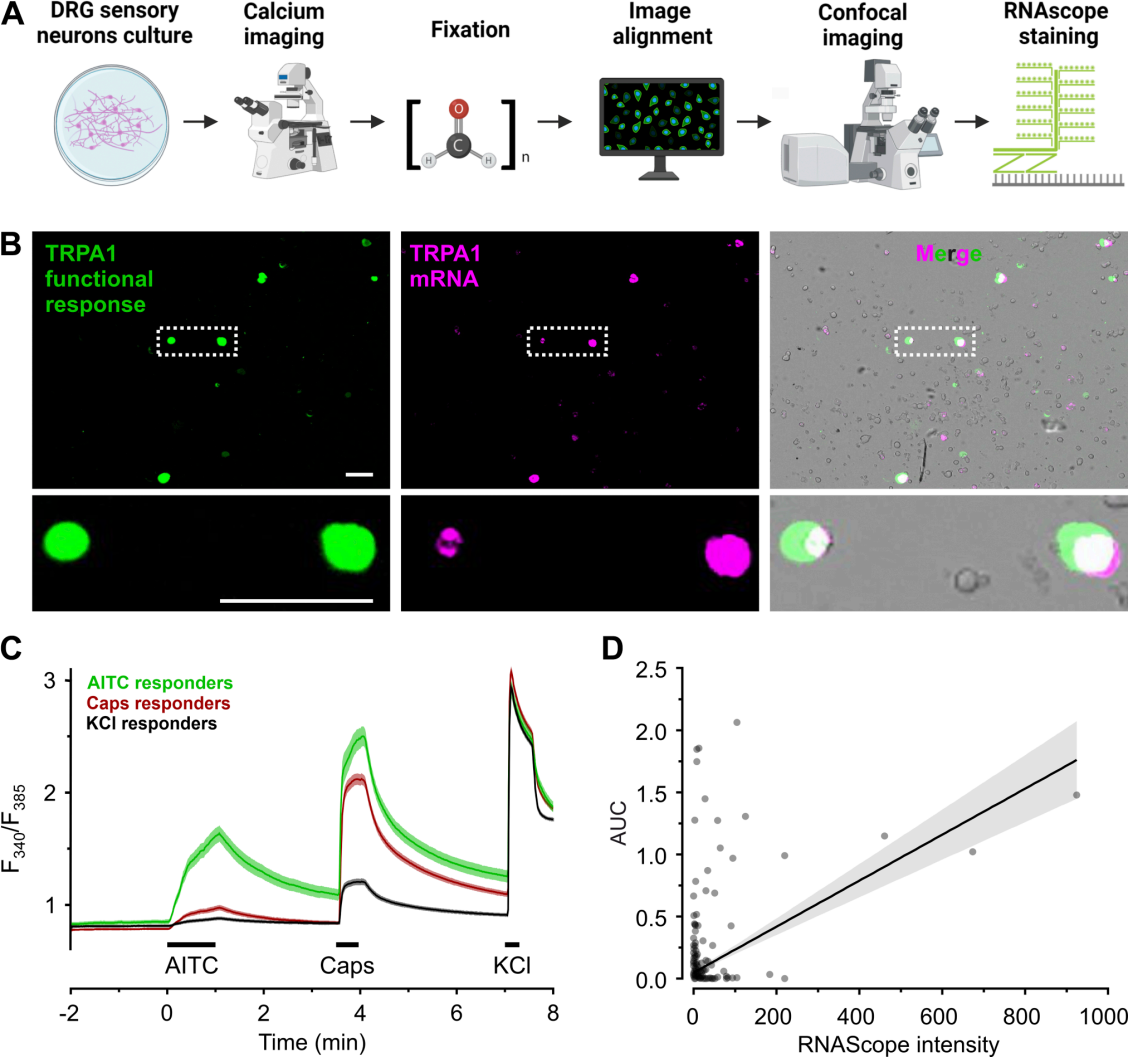
RNAscope for TRPA1 positively correlates to TRPA1-mediated calcium entry in mouse DRGs. A. Staining and imaging workflow to obtain aligned images of functional response and RNAscope signal. B. Representative images of the TRPA1 functional response (area under the curve coded in green), TRPA1 mRNA (magenta) and the overlay with transmitted light (gray) in cultured mouse DRGs. Enlarged image of neurons (dashed white box) with colocalized signal from TRPA1 functional response and mRNA (white). Scale bars = 100 μm. C. Cultured mouse DRG neurons were challenged with AITC 100 μM, capsaicin 1 μM and KCl 60 mM sequentially. Black bars indicate application periods. Cells that responded to KCl 60 mM and presented neuronal morphology were considered DRG sensory neurons. D. Pearson correlation *R* = 0.43 for 502 neurons with either a TRPA1 mRNA signal or a functional response to AITC 100 µM.

The same cultured DRG sensory neurons were first functionally tested, and immediately fixed with PFA 4% to detect mTRPA1 or mTRPV1 mRNA (Fig. 3A and B). For functional assessment, cells were loaded with Fura-2 and exposed to AITC 100 μM for 60 sec, capsaicin 1 μM for 30 sec and KCl 60 mM for 15 sec (Fig. 3C). RNAscope images aligned with functional images showed a positive association between calcium influx upon TRPA1 activation and mTRPA1 probes (*R* = 0.43, *p*<0.001, *n*=502, Fig. 3D). It should be noted that from 890 cells, 309 had an RNAscope signal without a functional signal, 95 had a functional signal without RNAscope signal and 98 were positive on both criteria.

**Figure A4.**
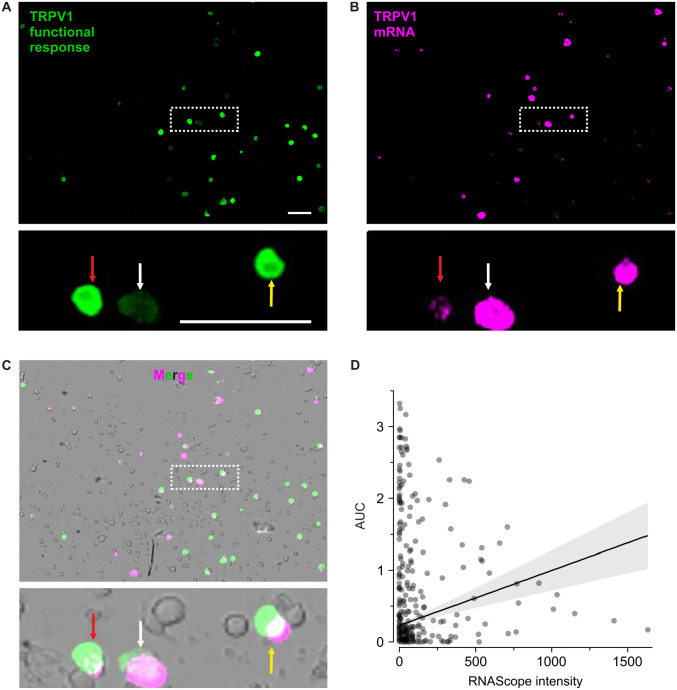
RNAscope for TRPV1 positively correlates to TRPV1-mediated calcium entry in mouse DRGs. A-C. Representative images of TRPA1 functional response (area under the curve for every pixel coded in green), TRPV1 mRNA (magenta) and the overlay with transmitted light (gray) in cultured mouse DRGs. Enlarged image of neurons (dashed white box) with colocalized signal from TRPV1 functional response and mRNA (yellow arrow), and cells with a predominantly functional or RNAscope signal (red and white arrow, respectively). Scale bar = 100 μm. D. Pearson correlation *R* = 0.18 for 833 neurons with either a TRPV1 mRNA signal or a functional response to Capsaicin 1 µM.

In addition, for TRPV1, the overlap of RNAscope with the response to the agonist capsaicin was evaluated. A subpopulation of neurons exhibited an overlap between TRPV1 functional response upon capsaicin 1 μM stimulation and TRPV1 mRNA. From a total of 890 cells investigated, 488 had a RNAscope signal without a functional signal, 22 had a functional signal without RNAscope signal, and 323 were positive on both criteria. Due to the substantial populations without matching RNAscope and functional signal, the correlation of *R* = 0.19 questions the use of these RNA probes as an index of functional TRPV1 (*p*<0.001, *n*=833, Fig. A4).

## Discussion

This study shows a positive correlation between TRPA1 detection by RNAscope and the well-established pharmacological agonist allyl isothiocyanate. The positive correlation is not as robust as anticipated, but it provides an index for functional TRPA1, which antibody-based techniques did not. Sensitivity is sufficient to detect mRNA in neurites.

Antibodies are a standard technique for determining expression levels and patterns. Researchers commonly bear the responsibility of validating commercial antibodies before using them, and there is no guarantee of their specificity. There appear to be proteins that, despite considerable effort, have eluded the generation of suitable antibodies. This is particularly problematic in the TRP channel field, as previously reported.^14,20^ Currently, https://www.biocompare.com/ lists 26 suppliers providing 117 antibody products against mouse TRPA1.

TRPA1 knockout animals are available in many laboratories. Pharmacological tools are not perfect, but provide a clear-cut picture in wild type versus TRPA1 knockout animals for several established substances, which allows testing of antibody-based results. After our own encounter with this issue,^13^ we decided to search for alternative tools that could provide a reliable and specific signal without compromising the detection sensitivity. Over the past years, the in situ hybridization-based technique RNAscope has been increasingly used in research and clinical studies due to its sensitivity and specificity, which have been evaluated in the context of pathologic diagnostics.^21–23^ RNAscope has been used to evaluate TRPA1 and TRPV1 mRNA abundance in neuronal cells from the peripheral^24–26^ and the central nervous system,^27–29^ as well as non-neuronal tumor cells.^30^ However, whether there is a correlation between mRNA and functional responses has not been assessed before.

A substantial overlap of mTRPA1 RNAscope and YFP fluorescent signals was observed in HEK293t cells co-transfected with murine TRPA1 and CFP::YFT, suggesting a considerable RNAscope specificity and sensitivity in a simplistic system. In about 27% of YFP-positive cells no RNAscope signal was detected, which may be attributed to the higher transfection efficacy of a smaller protein and the larger amount of CFP::YFP cDNA compared with mTRPA1. This choice was made to test the specificity of the RNAscope. The negligible fraction of TRPA1 RNAscope-positive and YFP-negative cells, in addition to the lack of RNAscope signal in non-transfected and mock-transfected HEK293t cells, support a high specificity, attributed to the design of the Z-probe dimers.^15,16^ RNAscope staining of TRPA1 and TRPV1 mRNA in cultured DRG sensory neurons allowed the detection of individual mRNA molecules with the characteristic punctate pattern. Signals were not only present in DRG sensory neurons perikarya, but also in neurites, most likely corresponding there to single RNA molecules. This does not allow the evaluation of sensitivity of the method, as the fraction of undetected mRNAs is unknown. Transport of mRNA to the axon terminals has been first described some time ago and is an established finding.^31–33^ Therefore, this should also be assumed for TRPA1 mRNA transport, given that there is evidence for TRPV1 undergoing axonal transport.^34^ Transport of mRNA indicated that translation is not exclusive to the perikarya. Adult mammalian DRG neurons locally synthesize structural proteins in the axon, at least under certain conditions such as after axonal crush injury.^35^ Thus, the observation of mRNA in neurites in this study, as a prerequisite for translation, aligns with prior literature on axonal transport. Furthermore, it might be speculated that these mRNAs are also peripherally maintained for a regulated on-demand translation. This might be an example for an investigation on mRNA level, and protein detection would only provide indirect evidence.

To evaluate RNAscope performance versus functional responses, cultured sensory neurons were acutely exposed to their respective agonists and then immediately stained with the corresponding RNAscope probes. Calcium responses upon TRPA1 activation and the signal from murine TRPA1 probes were positively correlated. However, a relevant amount of DRG neurons had a negligible response to AITC but a high TRPA1 probe intensity, and vice versa. Overall, both measures serve as a less definitive index of each other than anticipated, and over-interpretation should be avoided.

We also evaluated the performance of RNAscope in detecting functional TRPV1. The aim was to obtain additional data of RNAscope sensitivity and specificity in detecting the two channels, which have overlapping expression^5,9^ and are also functionally linked.^36,37^ Unexpectedly, there was only a marginal correlation between mRNA and functional TRPV1. Despite the sizable population of neurons exhibiting an overlap between TRPV1 functional response and mRNA signal intensity, two additional populations of cells either had a high functional intensity and no RNAscope signal, and vice versa, a clear RNAscope signal but no functional activity.

In this context, also the mismatch of fractions reported by single-cell RNAseq^38,39^ and functional data requires discussion. In particular, RNAseq reports that TRPA1 is expressed in a substantially larger fraction of neurons^38^ than functional experiments.

The recently published reporter mouse TRPA1^Flp/+^R26^ai65f/+^ allowed TRPA1 mapping by the endogenous expression of tdTomato in a flippase (Flp) sensitive manner.^9^ In examining the correlation between tdTomato expression and TRPA1 mRNA in the reporter mouse only 15.3% of AITC-responsive thoracic neurons were tdTomato positive. This mismatch was observed in intact ganglia, with 32–47% AITC-sensitive neurons versus 5–12% tdTomato positive neurons. There is a non-negligible rate of false-positives and false-negatives observed in the TRPA1^Flp/+^R26^ai65f/+^ mouse. Treatment with AITC generated calcium responses in 83% of tdTomato positive cells and in 27% of tdTomato negative cells, indicating a good but far from perfect correlation. Similarly, in our study the RNAscope signal has a substantial positive correlation to AITC-evoked responses. Therefore, both methods can serve as an index of the expected results of the other, but less so than what might have been expected. Despite the advantages of the TRPA1 reporter mouse, the period and effort to obtain it might provide some use cases for RNAscope, and further questions specifically addressing the RNA level, as mentioned above.

In this study, the fraction of neurons with RNAscope signal and without a functional signal is larger than vice versa. This might be due to a higher sensitivity but also due to a lower specificity, reporting false-positives. Intracellular microfluorimetric calcium detection is sensitive, and a just detectable signal might still be functionally irrelevant, while a functional relevant signal will not have gone unnoticed. Given the far higher rate of cells reported positive by RNAscope, the latter seems more likely to be overreporting TRPA1, than the calcium imaging to be underreporting.

As potential limitations of this study, the correlation between TRPA1 mRNA and functional protein has been only demonstrated in one expression system and in cultured DRG sensory neurons. It has been reported that the dissociation of sensory neurons leads to a disturbance of the transcript expression and electrophysiological properties.^40^ This calls for further testing in tissue sections and native tissue, which would be a potential application of RNAscope. However, for tracing axons of neurons which contain TRPA1, the mRNA levels seem to be too low, and the resulting signal too sparse. This might be overcome with axonal staining, connecting the sparse RNAscope signals. Furthermore, colocalization at a defined site, for example with markers of axon terminals, could be investigated. The nucleotides 77-1047 of mouse TRPA1 are the target for the 20 Z-primers used in this study. With 5.2% (51/970) nucleotides being different in human TRPA1 compared with mouse TRPA1, a large fraction of primers will not bind properly, asking for a separate primer design. As such, RNAscope for human TRPA1 is a different assay, and a similar correlation between hTRPA1 RNAscope and agonist responses in humans is hypothetical.

RNAscope is an increasingly popular technique and has already been used for mouse TRPA1. However, validation against another widely used functional method is lacking for many targets. Our study investigated this for TRPA1 and found a positive association, making TRPA1 RNAscope an additional tool, suitable to address specific questions.
